# Single‐Cell Dissection of the Biological Function and Molecular Features Underlying the Micropeptide LSMEM1 in Kidney

**DOI:** 10.1002/advs.202507713

**Published:** 2025-08-27

**Authors:** Peimin Liu, Hong Zhang, Shanzhi Yang, Jiaoqing Li, Danfeng Wu, Haosen Xu, Huan Jiang, Yi Jing, Guoxiang Jin, Ruobing Liu, Ning Fan, Xiaoyan Bai

**Affiliations:** ^1^ Department of Nephrology Guangdong Provincial People's Hospital (Guangdong Academy of Medical Sciences) Southern Medical University Guangzhou 510080 China; ^2^ Guangdong‐Hong Kong Joint Laboratory on Immunological and Genetic Kidney Diseases Guangdong Provincial People's Hospital (Guangdong Academy of Medical Sciences) Southern Medical University Guangzhou 510080 China; ^3^ Guangdong Cardiovascular Institute Guangdong Provincial People's Hospital Guangdong Academy of Medical Sciences Guangzhou 510080 China; ^4^ Suzhou Medical College Soochow University Suzhou 215123 China

**Keywords:** LSMEM1, micropeptide, single cell RNA‐seqencing

## Abstract

Advances in computational biology and large‐scale transcriptome analysis have revealed an increasing number of short open reading frames (sORFs) encoding functional peptides. These small proteins or micropeptides can function independently or exert their biological functions by binding to and/or regulating larger regulatory proteins. LSMEM1 (leucine rich single‐pass membrane protein 1, also known as C7orf53) has been found to be significantly upregulated in chronic kidney disease (CKD). In the present study, the molecular structure is aimed to elucidate and function of LSMEM1 and dissect its implications both in physiological and pathophysiological condition. Single‐cell transcriptome sequencing (scRNA‐seq) technology is used to examine the transcriptional state and biological processes of Lsmem1^−/−^ mice kidneys. Experiments are conducted to verify these biological processes in both physiological and disease states. LSMEM1 is associated with cell injury, inflammation and lipid metabolism. Further, LSMEM1 plays a critical role in delaying CKD progression through regulating lipid droplet accumulation in proximal tubular epithelial cells. This study explores the function of the small protein LSMEM1 in physiological and disease development. LSMEM1 may be a novel revenue for targeted therapy for CKD.

## Graphical abstract

LSMEM1, an evolutionarily conserved micropeptide with extreme hydrophobicity (aliphatic index═113) and dynamic amphiphilicity (GRAVY═0.017), features a strong α‐helical transmembrane anchor (residues 64‐86). Single‐cell analysis reveals its critical role in renal lipid homeostasis. The leucine‐rich membrane protein modulates tubular lipid droplet accumulation, linking its unique biophysical properties to chronic kidney disease progression and potential therapeutic targeting.

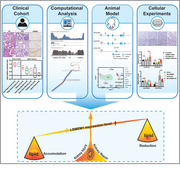

## 1 Introduction

Small proteins or micropeptides play an important role in disease occurrence, cell signaling and metabolic regulation. Micropeptides are generally defined as peptides of relatively arbitrary amino acid types, typically less than 100–150 amino acids in length.^[^ ^1^, ^2^ ^]^ Micropeptides act as peptide switches, directly or indirectly blocking ribosomes via small molecule activation, thereby inhibiting the expression of downstream coding sequences^[^ ^3^ ^]^. Despite being overlooked in some sequencing technologies, micropeptides are increasingly recognized as a significant portion of the genome being transcribed and translated, as technology advances. Many RNA sequences previously labeled as non‐coding actually contain short open reading frames (sORFs) encoding functional peptides. These proteins encoded by sORF can function independently or indirectly by binding to larger proteins and modulating them.^[^ ^1^ ^]^ Because of their small size, micropeptides may be well suited for regulating the body's complex biological systems. Additionally small molecules like these are also drawing attention in drug discovery. With the improvement of technology, an increasing number of small ORFs (sORFs) have been discovered, and the functions of these small proteins encoded by sORFs in physiological or disease states have garnered growing attention.

LSMEM1 (leucine rich single‐pass membrane protein 1, also known as C7orf53) is a class of small proteins with 133 amino acids that, to our knowledge, has not been previously recognized due to limitations in technical means. The aliphatic index of LSMEM1 is 113, indicating extreme hydrophobicity, while its GRAVY (grand average of hydropathicity) score of 0.017 suggests dynamic amphiphilic. Bioinformatic analysis predicts a transmembrane domain (residues 64–86) with strong α‐helical propensity, suggesting its role as a dynamic amphiphilic membrane anchor. Previous studies have shown that LSMEM1 is up‐regulated in atherosclerotic heart disease, psoriasis,^[^ ^4^ ^]^ prostate cancer^[^ ^5^ ^]^ and Parkinson's disease,^[^ ^6^ ^]^ and some studies have found that LSMEM1 gene mutations may drive primary bladder signet ring cell carcinoma.^[^ ^7^ ^]^ However, the mechanism of LSMEM1 up‐regulation in diseases has not yet been comprehensively reported.

To understand the role of LSMEM1 in kidney disease, we designed the present study to elucidate its potential function in both physiology and disease state. Single‐cell RNA sequencing techniques were employed to elucidate the biological processes in which LSMEM1 may be involved at the transcriptional level.

## 2 Results

### 2.1 Up‐Regulation of LSMEM1 During the Development of Disease

**Figure 1:**
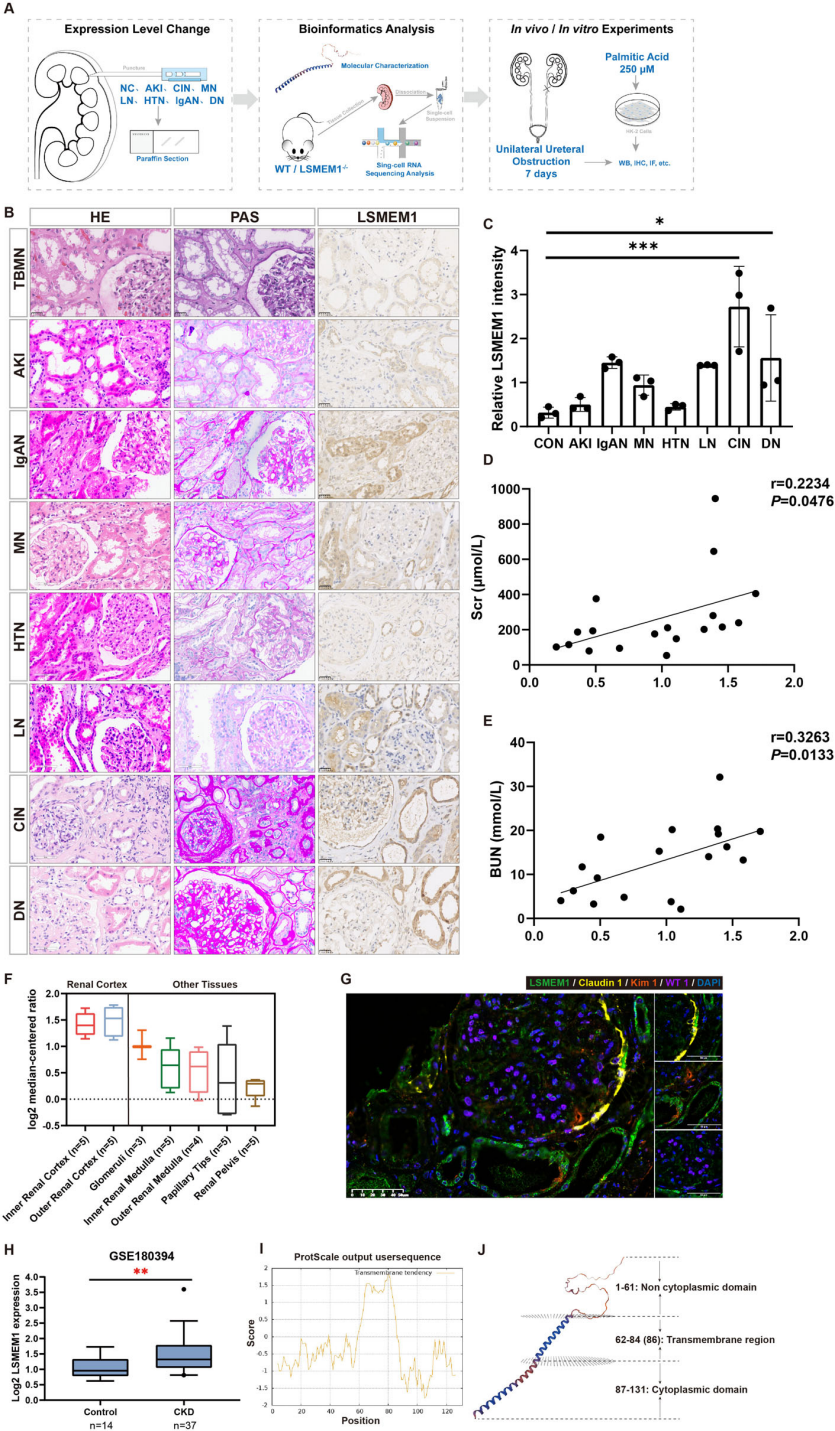
The expression level, location, and molecular model of LSMEM1 in the human kidney. A) study design. B) LSMEM1 expression was visualized by immunohistochemistry, and HE and PAS staining were performed to visualize the damage of the kidney. C) Quantification of LSMEM1 expression (*n* = 24, thin‐basement membrane nephropathy (TBMN) and normal kidney tissues distant from kidney stone resection surgery served as controls (*n* = 3), AKI (*n* = 3), and CKD (*n* = 18, including LN, IgAN, CIN, MN, DN, and HTN); One‐way ANOVA test. D) The correlation of LSMEM1 expression and Cre (*n* = 18, Pearson χ2 test. E) The correlation of LSMEM1 expression and BUN (*n* = 18, Pearson χ2 test. F) Expression of LSMEM1 in different parts of the kidney tissue. Data analysis from the Nephroseq database (median‐centered log2). G) Representative images of Immunostaining of LSMEM1, KIM1, WT1, and Claudin1 in human kidney tissues. Scale bars = 50 µm. Proximal tubule cells were labeled by KIM1. Podocytes were labeled by WT1. Parietal epithelial cells were labeled by Claudin1. H) The mRNA levels of LSMEM1 in kidney specimens of CKD (*n* = 37 samples) and control (*n* = 14 samples) in the GSE180394 dataset. Unpaired t‐test. I) Expase prediction showed that LSMEM1 has a transmembrane region. J) Predictive molecular model of LSMEM1. AKI: acute kidney injury; LN: lupus nephritis; IgAN: IgA nephropathy; CIN: chronic interstitial nephritis; MN: membranous nephropathy; DN: diabetic nephropathy; HTN: hypertensive nephropathy. * *p* < 0.05, ** *p* < 0.01, *** *p* < 0.001.

To better understand the function of LSMEM1, we first tested LSMEM1 expression levels in patients and mice during disease progression. We then performed single‐cell RNA sequencing (scRNA‐seq) on kidney samples from wild‐type (WT) mice (*n* = 4) and LSMEM1 knockout (KO) mice (*n* = 4). Finally, we verified LSMEM1 function through experiments (**Figure** **1**A). We analyzed human kidney samples from patients with acute kidney injury (AKI) and CKD. Clinical demographics of these subjects were provided in Table S1 (Supporting Information). Patients with AKI and CKD exhibited severe kidney injury as detected by hematoxylin eosin (HE) staining and periodic acid schiff (PAS) staining (Figure 1B). LSMEM1 protein levels were significantly higher in patients with CKD (especially in chronic interstitial nphritis and diabetic nephropathy group) compared to normal controls as detected by immunohistochemical staining (Figure 1B,C). Furthermore, LSMEM1 levels were positively correlated with serum creatinine (Figure 1D) and blood urea nitrogen (Figure 1E).

Next, we set out to examine the location and expression level of LSMEM1 through analyzing the renal transcriptomics database. Through analysis of the Nephroseq database we found that LSMEM1 expression was primarily localized to the renal cortex of the kidney (Figure 1F). Through multiplex immunofluorescence labeling, we further confirmed the cellular localization of LSMEM1 in CKD patients by assessing the marker genes in podocytes, parietal epithelial cells and proximal tubule epithelial cells (Figure 1G). Similar to the results as shown in the immunohistochemical staining of LSMEM1, GEO database (Figure 1H) showed LSMEM1 mRNA levels were significantly higher in CKD patients.

To comprehensively model human kidney diseases, we employed ischemia/reperfusion injury (IRI) and LPS (Lipopolysaccharide)‐induced sepsis for acute kidney injury (AKI), db/db and streptozotocin (STZ) models for diabetic nephropathy, hypertensive nephropathy (HTN) models, and unilateral ureteral obstruction (UUO) and folic acid (FA) models for tubulointerstitial fibrosis (Figure S1A–C, Supporting Information). Our results indicated that LSMEM1 expression was increased during kidney disease progression. Biological parameters of mice are shown in Table S2 (Supporting Information). The location of Lsmem1 in mice kidneys were detected using multiplex immunofluorescence staining with marker genes for podocytes, endothelial cells and proximal tubular epithelial cells (FigureS 1D,E). The results have shown that Lsmem1 was localized in cytoplasm of podocytes, parietal epithelial cells and proximal tubule epithelial cells. Interestingly, we found that Lsmem1 was also located within the nucleus.

### 2.2 Structural Features of LSMEM1

Bioinformatic analysis revealed that LSMEM1 is a small protein composed of 133 amino acids, and mouse Lsmem1 is homologous to human. The molecular characteristics of LSMEM1 in both humans and mice were demonstrated in Table S3 (Supporting Information). LSMEM1 exhibits a high aliphatic index yet a near‐neutral GRAVY value, suggesting its potential as an amphipathic molecule capable. Expase predicts a transmembrane region in LSMEM1 (Figure 1I), while integrated InterPro/PSIPRED/SwissModel analyses reveal its structural pattern (Figure 1J). To predict LSMEM1's molecular structure, we employed multiple bioinformatic tools (FuzDrop method,^[^ ^8^, ^9^, ^10^ ^]^ P2D2 database^[^ ^11^ ^]^ and NLSExplorer^[^ ^12^ ^]^) (Figure S2, Supporting Information). Our analyses reveal that LSMEM1 functions as a dynamic amphiphilic membrane anchor, featuring disordered regions with protein‐binding capacity. These structural properties enable conformational plasticity, facilitating transitions between disordered and ordered binding modes to mediate diverse molecular interactions (Figure S2A–D, Supporting Information). This has important implications for the functions that proteins may perform. Furthermore, we also used bioinformatic method Cell‐PLoc 2.0^[^ ^13^ ^]^ to predict the localization of this uncharacterized protein (Table S4, Supporting Information). Similar to our experimental results, the analytic data also reveal that it may localize to the cytoplasm and nucleus. Subsequently, using the NLSExplorer online tool, we predicted the nuclear localization sequences (NLS, Figure S2E, Supporting Information) and the potential segment important for nuclear transport (Table S5, Supporting Information).

### 2.3 Single‐Cell Transcriptome Landscape of Lsmem1^−/−^ Mice Kidney

To better explore the function of LSMEM1, we constructed Lsmem1 knockout (KO) mice for transcriptome analysis and identified the genotype of mice (Figure S3, Supporting Information). In accordance with the omics study sample preparation standards, we collected mice kidneys for single‐cell transcript omic analysis.

**Figure 2:**
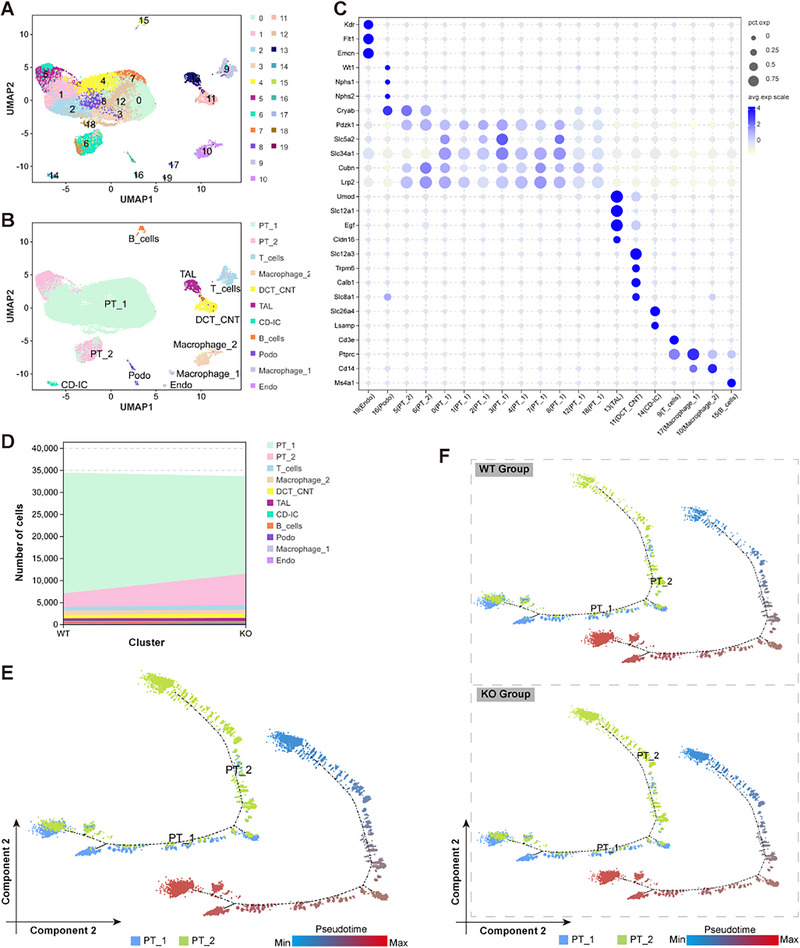
scRNA‐seq profile of kidney from WT and Lsmem1^‐/‐^ mice samples. A) UMAP plots colored by different cell clusters. B) UMAP plots show the different cell types in the kidney. C) Dot plots of scRNA‐seq dataset showing gene expression patterns of cluster‐enriched markers. The diameter of the dot corresponds to the proportion of cells expressing the indicated gene, and the density of the dot corresponds to average expression relative to all cell types. D) The proportions of each cell type in the WT and Lsmem1‐/‐ groups. E) Pseudotime trajectory analysis corresponding to the PT cell subclusters using Monocle 2. Cells are colored by pseudotime or cell type. F) Pseudotime trajectory analysis corresponding to the PT cell subclusters just in the WT or Lsmem1^‐/‐^ group using Monocle 2. Cells are colored by cell type or pseudotime.

The dimension reduction distribution map of renal cell clusters using UMAP, showed that cells in kidney tissues are divided into 20 clusters according to transcriptome characteristics (**Figure** **2**A). Using R‐based single‐cell analysis, we annotated cell subsets according to canonical markers in Table S6 (Supporting Information), identifying 9 major renal cell types: podocytes (PODO), endothelial cells (ENDO), proximal tubule cells (PT; subdivided into PT1 and PT2 subpopulations based on the discovery of a parietal epithelial cell marker‐expressing subset, see Figure S4B, Supporting Information), thick ascending limb (TAL), distal convoluted tubule/connecting tubule (DCT_CNT), collecting duct‐intercalated cells (CD_IC), and immune cells (Figure 2B,C). The relative abundance of major cell populations in WT and Lsmem1^−/−^ samples (Figure 2D, Figure S4A,B, Supporting Information) showed an increased proportion of PT2 subsets in the LSMEM1 group compared with the WT group. We subsequently used the scRNA‐seq data and investigated the relationship of PT1 and PT2 clusters at single‐cell resolution using Monocle analysis for trajectory inference. Pseudotime trajectory analysis (Figure 2E) revealed a potential transition from PT1 to PT2, suggesting cellular state progression. Notably, Lsmem1 knockout (KO) mice exhibited a significant increase in PT2 population frequency compared to wild‐type (WT) controls (Figure 2F), implicating Lsmem1 in regulating tubular cell state plasticity. Data S1 (Supporting Information) presents a total of 6919 DEGs (differentially expressed genes) between PT1 and PT2 with pseudotime analysis.

We also analyzed the number of upregulated genes. All subpopulations showed upregulation, with the most prominent in the PT2 group, followed by the PT1 and PODO groups. Gene Ontology (GO) analysis revealed PT2 up‐regulated genes were enriched in biological processes, while PT1 and PODO showed cellular component enrichment (Figure S4C–E, Supporting Information). Kyoto Encyclopedia of Genes and Genomes (KEGG) analysis indicated PT2 genes were metabolism‐associated, whereas PT1 and PODO genes were linked to junction/cytoskeleton pathways (Figure S4F–H, Supporting Information).

### 2.4 Transcriptome Signature of PT Cell Clusters in Lsmem1 Knockout Mice

In Lsmem1 knockout mice, we focused on two subpopulations of renal proximal tubules (PT), PT1 and PT2, for their most noticeable change after the Lsmem1 knockout. To facilitate biological understanding of PTs, DEGs of subpopulations were analyzed in terms of GO and KEGG analysis.

**Figure 3:**
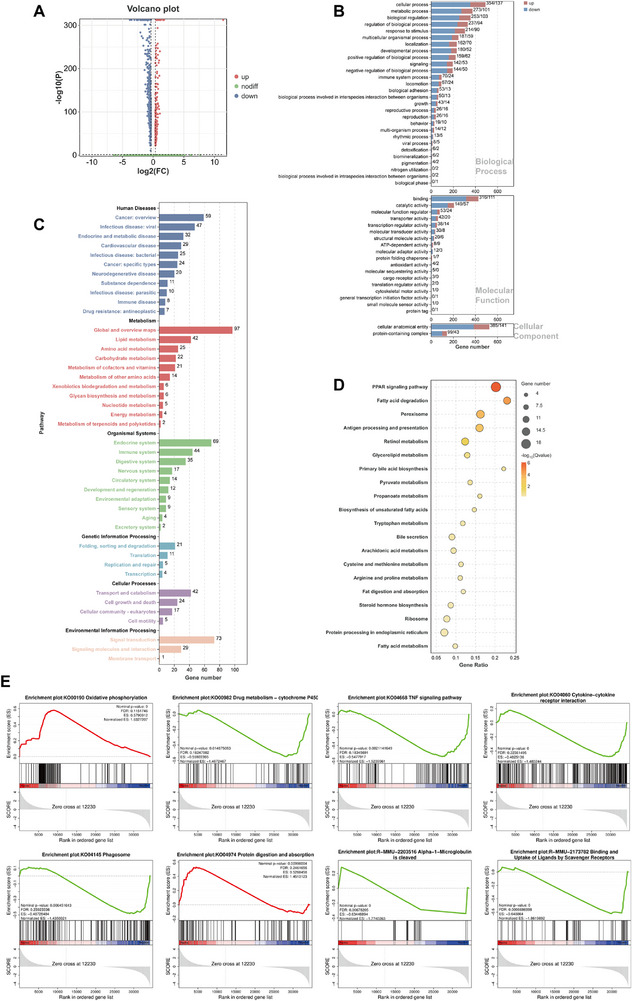
Characteristics of PT1 clusters in Lsmem1^‐/‐^ mice. A) Heat map shows the total 608 DEGs between WT and Lsmem1‐/‐ mice. GO B) and KEGG C) enrichment results of DEGs presented by a bar chart. The x‐axis represents gene numbers, and the y‐axis represents GO/KEGG terms. D) Dot plots exhibited the top 20 KEGG pathways of DEGs. The x‐axis represents gene ratio, and the y‐axis represents KEGG pathways. The size of the circle represents gene count. Different color of circles represents the Q value. E) GSEA analysis showing the representative KEGG/Reactome pathways in the Lsmem1‐/‐ group.

Our analyses of the 608 DEGs (**Figure** **3**A, Data S2, Supplementary Data) in the PT1 group revealed enrichment in cellular processes, binding, and cellular anatomical entities via GO analysis (Figure 3B), while KEGG pathways highlighted lipid metabolism, carbohydrate and amino acid metabolism, and endocrine/immune system regulation (Figure 3C), with specific lipid‐related pathways (Figure 3D, Data S3, Supplementary Data) such as PPAR signaling, fatty acid degradation, and peroxisome activity underscoring LSMEM1's potential role in renal lipid regulation. GSEA analysis (*p *< 0.05, FDR<0.25; 239 GO, 27 KEGG and 71 Reactome terms showed in Data S4–S6, Supplementary Data) comparing Lsmem1^−/−^ and WT mice further supported these findings, showing significant enrichment in oxidative phosphorylation and protein digestion/absorption (positive NES) alongside suppressed pathways like drug metabolism–cytochrome P450, TNF signaling, cytokine interactions, and phagosome activity (negative NES) in Figure 3E. The prominence of lipid metabolism pathways—particularly PPAR signaling, where CD36, a key scavenger receptor, was differentially expressed—reinforced LSMEM1's involvement in metabolic regulation. Additionally, Reactome analysis (Data S6, Supplementary Data) linked Lsmem1 not only to immune and complement pathways but also to lipid‐related processes such as alpha‐1‐macroglobulin cleavage and scavenger receptor‐mediated ligand uptake, as well as STAT‐dependent transcriptional regulation, collectively implicating LSMEM1 in lipid homeostasis, inflammation, and cellular injury responses in renal proximal tubules.

**Figure 4:**
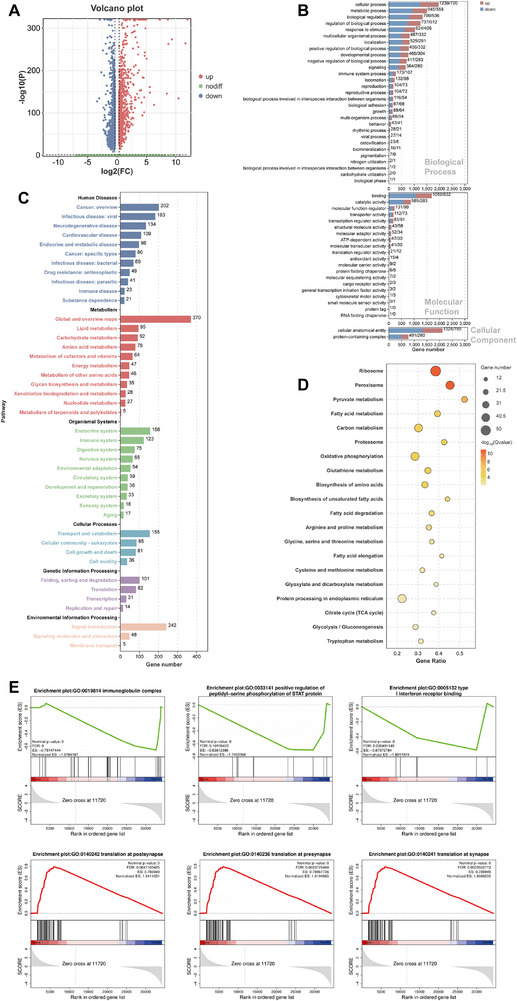
Characteristics of PT2 clusters in Lsmem1^‐/‐^ mice. A) Volcano plot reveals the total number of 2367 differentially expressed genes (DEGs) between wild‐type (WT) and Lsmem1‐/‐ mice. GO B) and KEGG C) enrichment results of DEGs presented by a bar chart. The x‐axis represents gene numbers, and the y‐axis represents GO/KEGG terms. D) Dot plots exhibited the top 20 KEGG pathways of DEGs. The x‐axis represents gene ratio, and the y‐axis represents KEGG pathways. The size of the circle represents gene count. Different color of circles represents the Q value. E) GSEA analysis showing the representative GO pathways in the Lsmem1‐/‐ group.

Similar GO and KEGG analyses of the PT2 cluster revealed enrichment in cellular processes, lipid metabolism, and immune pathways, mirroring PT1 findings (**Figure** **4**A–C, Data S7, Supplementary Data), but with additional energy metabolism pathways like oxidative phosphorylation, citrate cycle (TCA cycle) and glycolysis / gluconeogenesis (Figure 4D, Data S8, Supplementary Data), suggesting broader functional roles for Lsmem1 in PT2. GSEA analysis (Figure 4E, 7 GO and 81 Reactome terms showed in Data S9, S10, Supplementary Data) highlighted synaptic translation (positive NES) and immune‐related processes such as “immunoglobulin complex”, “type I interferon receptor binding” and “positive regulation of peptidyl‐serine phosphorylation of STAT protein” (negative NES), along with enriched complement pathways and specific involvement of eIF2ak4 and FGFR2 signaling, further linking Lsmem1 to cellular signaling and immune regulation.

These results denote the functional heterogeneity among PT subpopulations, suggesting Lsmem1 may be involved in maintaining normal biological processes. Whether the up‐regulation of Lsmem1 in the disease state exacerbates or alleviates changes in these processes needs further verification in a disease model.

Based on our experiments that LSMEM1 is also expressed in podocytes, we conducted a preliminary analysis of podocytes. The KEGG analysis results (Data S11, Supplementary Data) indicated significant enrichment in energy metabolism pathways, such as “oxidative phosphorylation” and the “apelin signaling pathway”. As this study most significantly impacted tubule cells, we primarily validated the role of LSMEM1 in tubule cells using in vitro and in vivo experiments.

### 2.5 Role of LSMEM1 in Lipid Metabolism of Renal Tubular Cells

**Figure 5:**
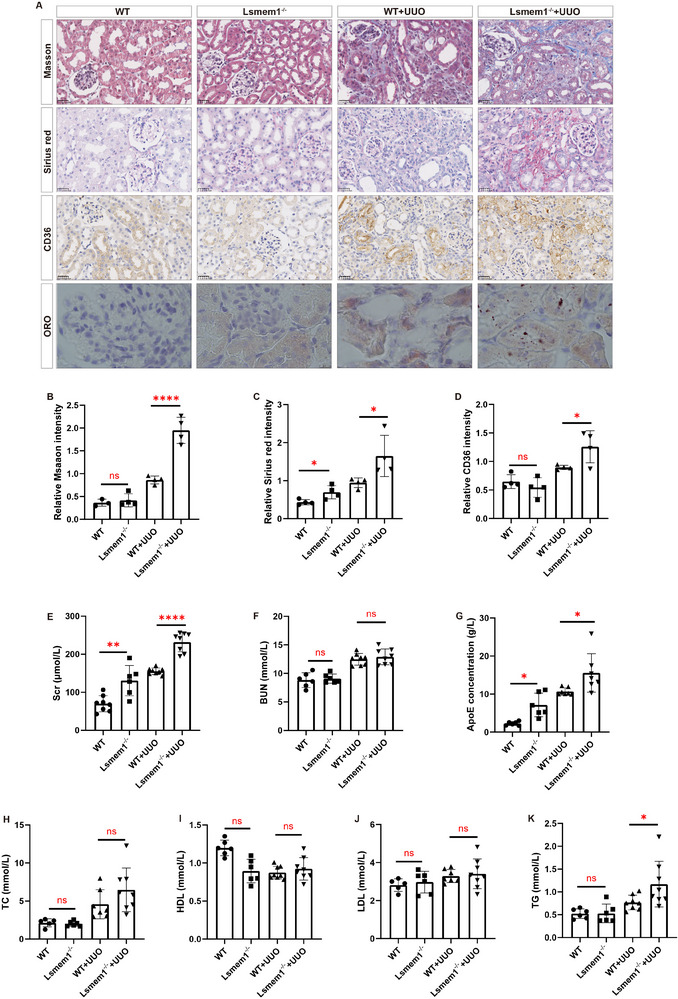
Knockdown of Lsmem1 aggravates the accumulation of lipid droplets in the kidneys of UUO mice. A) Masson trichrome and Sirius red staining were performed to visualize the damage to the kidney. CD36 expression was visualized by immunohistochemistry, and ORO staining was performed to visualize the lipid droplets. Quantification of Masson trichrome B) and Sirius red staining C) in kidneys (*n* = 4). D) Quantification of CD36 expression (*n* = 4). The Scr E) and BUN F) levels of serum. G–K) The TG, TC, LDL, HDL, and ApoE levels of serum in the WT and Lsmem1‐/‐ mouse kidneys at day 7 after UUO. All data are presented as means ± s. e.m, and * *p* < 0.05, ** *p* < 0.01, *** *p* < 0.001, **** *p* < 0.0001. TG: triglyceride; TC: cholesterol; LDL: low‐density lipoprotein; HDL: high‐density lipoprotein; Scr: serum creatinine; BUN: urea nitrogen.

Our results have shown that LSMEM1 was most prominently up‐regulated in CKD patients and CKD animal models, and lipid‐associated pathways were significantly enriched in the PT cluster. Therefore, we used UUO mice and PA‐stimulated HK‐2 cells to validate the role of LSMEM1 in lipid metabolism in proximal tubule cells. Upon Lsmem1 knockout, increased fibrosis was detected as demonstrated by Masson trichrome and Sirius red staining (**Figure** **5**A–C). Biological parameters of mice are shown in Table S2 (Supporting Information). In Lsmem1^−/‐^ mice, elevated expression of CD36 (also known as scavenger receptor B2, Figure 5A,D) and enhanced accumulation of lipid droplets (Figure 5A) were shown as examined by immunohistochemistry and oil red O (ORO) staining, respectively. As tested by the biological function analysis in mice, the level of serum creatinine was increased in Lsmem1^−/‐^ mice (Figure 5E), while the urea nitrogen level remained unchanged (Figure 5F). Further, we measured lipid metabolism related indexes in the serum and kidney of mice, and the results showed that in Lsmem1^−/‐^ mice, the levels of the serum and kidney triglycerides, cholesterol, and low‐density lipoprotein were increased, while the high‐density lipoprotein level was decreased (Figure 5G–K).

**Figure 6:**
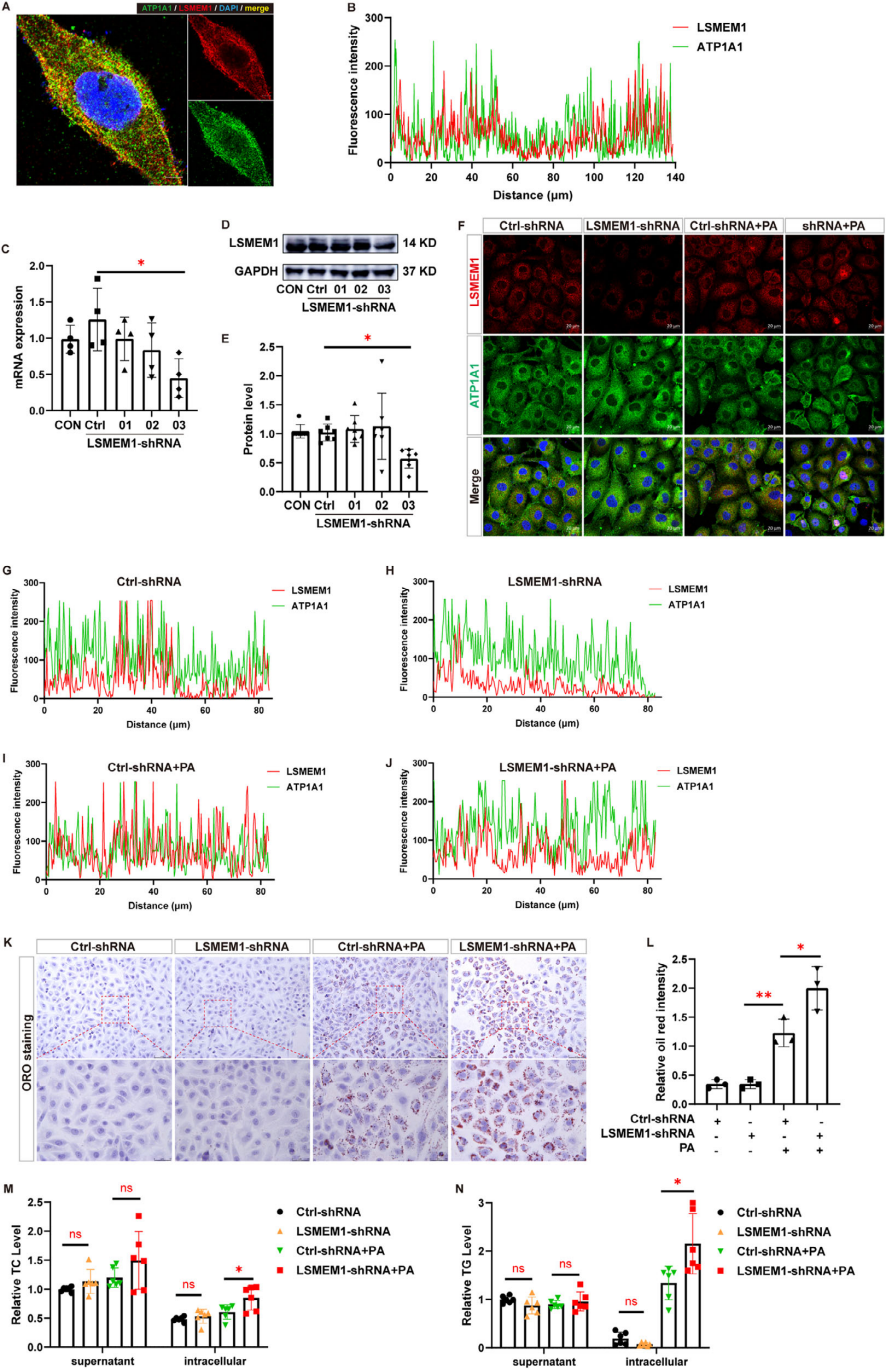
Knockdown of LSMEM1 aggravates the accumulation of lipid droplets in the HK‐2 cell after PA incubation. A) Representative images of immunostaining of LSMEM1 and ATP1A1 in HK‐2 cells. Scale bars = 10 µm. B) Fluorescence intensity of LSMEM1 and ATP1A1; Image J software was used for statistics. Scale bars = 20 µm. C) mRNA expression of LSMEM1 in the LSMEM1‐silenced HK‐2 cells is measured by RT‐qPCR. D) Protein expression of LSMEM1 (sample volumes (40 µg protein/lane)) in the LSMEM1‐silenced HK‐2 cells is measured by western blotting, and semi‐quantitative data E) are presented. F) Representative images of immunostaining of LSMEM1 and ATP1A1 in the LSMEM1‐silenced HK‐2 cells after PA incubation for 48 h, and Fluorescence intensity data G–J) were presented. K) Lipid accumulation in the LSMEM1‐silenced HK‐2 cells after PA incubation for 48 h, and Quantitative data L) were presented. M, N) Relative TG and TC levels in cell supernatant and intracellular after LSMEM1 silencing. All data are presented as means as ± s.e.m, and * *p* < 0.05, ** *p* < 0.01, *** *p* < 0.001.

To elucidate the biological function of LSMEM1 in vitro, we used PA‐stimulated HK‐2 cells to validate the localization of LSMEM1 in HK2 tubule epithelial cells (**Figure** **6**A,B). The knocking down efficiency of LSMEM1 lentivirus silencing was verified by RT‐qPCR and Western blotting analyses (Figure 6C–E). The results have shown that the decreased expression level of LSMEM1 upon LSMEM1 silencing was recovered with PA stimulation (Figure 6F). Further, the cytoplasm to nucleus translocation was observed after PA stimulation (Figure 6F–J). Silencing LSMEM1 aggravated lipid droplet accumulation as shown by ORO staining (Figure 6K,L). TC/TG assay showed that the intracellular TC/TG level increased after LSMEM1 silencing, but had little effect on cell supernatant (Figure 6M,N).

Both the in vivo animal experiments and in vitro cell experiments have indicated that knockdown of LSMEM1 exacerbates the lipid accumulation in renal tubular cells during disease progression. In addition to knockdown of LSMEM1, an overexpression approach was performed using lentivirus to introduce LSMEM1 into cells (Figure S5, Supporting Information). Our results have revealed that overexpression of LSMEM1 attenuated lipid droplet deposition in renal proximal tubular cells under disease progression. LSMEM1 plays an important role in maintaining normal lipid metabolism.

## 3 Discussion

Traditional omics approaches typically prioritize longer ORFs (>300 nucleotides or 100 amino acids) and database‐matched abundant proteins, leading to systematic oversight of micropeptides due to both methodological biases and sample preparation limitations.^[^ ^1^, ^14^ ^]^ However, advancing technologies like RNA sequencing (RNA‐Seq) are now enabling comprehensive identification of small proteins by providing complete transcriptome profiles that include novel sequences.^[^ ^14^ ^]^

Our study revealed significant LSMEM1 upregulation in chronic kidney disease patients, highlighting the need to characterize this leucine‐rich single‐pass transmembrane micropeptide to elucidate its physiological and pathological roles.

Analysis of LSMEM1 reveals it is a 133‐amino acid micropeptide with distinct structural features, including a transmembrane alpha‐helical domain and an extracellular beta‐fold region, characterized by dynamic amphiphilic and leucine‐rich disordered/protein‐binding motifs. Like other micropeptides encoded by sORFs (<100–150 aa), its small size confers advantages such as high specificity and low cytotoxicity, enabling roles in complex processes like signal transduction and protein interactions.^[^ ^2^, ^15^ ^]^ As a single‐pass transmembrane protein (TMP), LSMEM1 differs from monomeric membrane‐anchored proteins by traverse the entire lipid bilayer, a feature typical of TMPs that function as receptors, transporters, or signal transducers, establishing electrochemical gradients and serving as biomaterials for sensors, water purification, and energy applications.^[^ ^1^, ^16^ ^]^ Studies of the effect of calcium ions on sodium‐potassium ATPase have shown that the expression of LSMEM1 increases significantly after treatment with sodium‐potassium ATPase inhibitors in endothelial cells. Researchers then used G4Catchall, an online tool to predict G‐quadruplex formation, and found that these structures may be formed in regions 1–16 of the LSMEM1 gene.^[^ ^17^ ^]^ The simplicity of its structure and extracellular domain along with their evolutionary history with the human body result in excellent selectivity for specific protein targets, making such TMPs valuable for drug development, including target prediction, TPD (Targeted Protein Degradation) technologies, and membrane‐protein interaction studies, with >50% of current drugs targeting TMPs.^[^ ^16^, ^18^, ^19^, ^20^ ^]^ While the leucine‐rich sequences in LSMEM1 remain unclassified, domains like leucine zippers and leucine‐rich repeat (LRR) are known to mediate cell adhesion, ligand binding, and synaptic signaling.^[^ ^21^, ^22^ ^]^ Its disordered regions suggest potential involvement in liquid‐liquid phase separation (LLPS) and membrane‐less organelle (MLO) formation, processes critical for cellular compartmentalization and signaling.^[^ ^23^ ^]^ Though no micropeptides have been directly linked to LLPS, their regulatory potential is significant.^[^ ^3^ ^]^

Multiple studies have demonstrated the role of LSMEM1 in neurological diseases. For example, LSMEM1 is most upregulated in the choroid plexus epithelium (CPE) of Alzheimer's disease patients compared to healthy CPE.^[^ ^24^ ^]^ In subarachnoid hemorrhage patients with delayed cerebral ischemia (DCI), LSMEM1 was enriched in the top 10 differentially methylated genes.^[^ ^25^ ^]^ In mouse cerebral cortex transcriptome sequencing, LSMEM1 was highly upregulated in treated epilepsy mice.^[^ ^26^ ^]^ LSMEM1 was also significantly upregulated in whole blood transcriptome sequencing of Parkinson's and Alzheimer's patients.^[^ ^6^, ^27^, ^28^ ^]^ Mutations in the LSMEM1 gene were found in genotyping and testing of autism patients.^[^ ^29^ ^]^ LSMEM1 is upregulated in a rat spinal cord pain model and enriched in differential genes for neurocytoma and accessory blastoma.^[^ ^30^, ^31^ ^]^ The second most common focuses on studies related to digestive system malignancies. For example, it is upregulated in colorectal cancer and pancreatic ductal carcinoma.^[^ ^32^, ^33^, ^34^, ^35^ ^]^ Studies have also shown that in patients with systemic sclerosis who develop pulmonary hypertension, patients with osteoarthritis, and cells infected with Cryptococcus neoformans H99 and Cryptococcus gattii R265, LSMEM1 is differentially enriched.^[^ ^36^, ^37^, ^38^ ^]^ These studies suggest that LSMEM1 may be involved in complex regulatory systems such as intercellular signal transduction or cytokine interactions due to its unique molecular structure. In addition, in peripheral blood sequencing, LSMEM1 was upregulated in fetuses delivered at full term compared with those not delivered at full term,^[^ ^39^ ^]^ which also suggests that LSMEM1 may play an important role in maintaining normal physiological functions.

Our study reveals that LSMEM1 is upregulated in chronic kidney disease (CKD), and its knockout or silencing exacerbates renal tubular injury. Similar to protective proteins like Nrf2—which is elevated in UUO models and human tubulointerstitial nephritis, with knockout worsening tubular damage and fibrosis^[^ ^40^ ^]^ —or NGAL, whose deficiency aggravates diabetic kidney injury by increasing oxidative stress and fibrosis.^[^ ^41^ ^]^ we propose that LSMEM1 may act as a renal protective factor. Under physiological conditions, it likely maintains homeostasis, while during disease, its upregulation could counteract injury progression through specific mechanisms.

Database analysis combined with our results demonstrate LSMEM1's predominant expression in proximal tubules, parietal epithelial cells (PECs), and podocytes. Although technical limitations prevented clear isolation of PECs in our single‐cell sequencing, observations from human disease models (showing significant LSMEM1 upregulation in diabetic kidney disease and tubulointerstitial nephritis) and animal studies (consistent elevation in diabetic nephropathy and fibrosis models) confirm its expression across these three cell types. These developmentally related cells – all derived from metanephric mesenchyme – maintain structural continuity through PEC‐podocyte connections at the glomerular vascular pole forming Bowman's capsule, and PT‐capsule linkages at the urinary pole, with disease states potentially inducing cellular transdifferentiation between them.^[^ ^42^, ^43^ ^]^ Our single‐cell RNA‐seq of LSMEM1 KO mice revealed its strongest functional impact on proximal tubule (PT) cells, particularly a PT2 subpopulation exhibiting PEC markers identified through Seurat/Monocle2 analysis,^[^ ^44^, ^45^, ^46^ ^]^ where knockout increased greater both PT2 proportion and upregulated genes compared to PT1. Given PECs‐related reported plasticity – containing podocyte/tubular progenitor subpopulations critical for crescent formation in anti‐GBM glomerulonephritis,^[^ ^43^ ^]^ LSMEM1 may mediate PEC‐tubular epithelial regulation, though this requires experimental validation. Given kidneys' high energy demands, with tubules preferentially utilizing fatty acids (FA) via transporters CD36 (a class B scavenger receptor and long‐chain FA transporter) and FATP2 (FA protein transporter 2) (both highly expressed in proximal tubular cells and podocytes, and are associated with lipotoxicity),^[^ ^47^ ^]^ these findings suggest LSMEM1's potential role in FA metabolism. Enriched terms like oxidative phosphorylation and apelin signaling in podocytes further support this connection, as apelin regulates renal hemodynamics, podocyte function^[^ ^48^, ^49^ ^]^ and mitochondrial metabolism^[^ ^50^ ^]^ while its deficiency causes FA dysregulation.^[^ ^48^ ^]^ The co‐enrichment of alpha‐1‐microglobulin (a lipocalin involved in renal oxidative stress)^[^ ^51^ ^]^ and lipid pathways highlights LSMEM1's potential involvement in both metabolic and oxidative stress regulation. Moreover, several pathways related to immune response, cytokine interaction, and synaptic translation were enriched, suggesting LSMEM1 may either directly participate in immune regulation or indirectly initiate immune activation through lipid oxidative stress mechanisms. For their small size enables multi‐pathway influence, a single micropeptide may concurrently affect metabolism, inflammation and apoptosis through distinct molecular interactions.^[^ ^52^, ^53^ ^]^ These findings potentially explain LSMEM1's upregulated expression patterns observed across various renal pathologies including IgA nephropathy, lupus nephritis, and LPS‐induced septic AKI, though its most pronounced effects appear in highly energy‐dependent cell types like tubular epithelial cells and podocytes.

As the first study to characterize this molecule, we focused our investigation on its most prominently expressed sites, ultimately selecting two representative models, the tubulointerstitial fibrosis model (UUO) and lipid metabolic model (PA‐stimulated HK‐2 cells), for detailed analysis. Our findings demonstrated that LSMEM1 shows predominant tubular localization and significant upregulation in kidney fibrosis, leading us to specifically examine its role in renal lipid metabolism. Functional studies revealed that LSMEM1 knockout or silencing exacerbated lipid droplet accumulation in both in vivo (UUO mice) and in vitro (PA‐treated HK‐2 cells) systems, while its overexpression conversely attenuated this lipid accumulation phenotype. Lipid droplet (LD) accumulation, a hallmark of CKD, represents both cellular dysfunction and potential protective mechanisms against lipotoxicity in renal cells.^[^ ^47^, ^54^ ^]^ LDs contain triacylglycerol/cholesteryl ester cores surrounded by phospholipid monolayers with two protein classes: Class I proteins (typically with hydrophobic membrane hairpins inserted in the endoplasmic reticulum (ER)) and Class II proteins (recruited directly from the cytosol to the LD surface).^[^ ^54^ ^]^ PPAR and peroxisomes play key roles in renal lipid metabolism, with tubules normally relying on fatty acid oxidation for energy.^[^ ^47^, ^54^, ^55^ ^]^ Given this structural context of lipid droplets, whether LSMEM1 – featuring a transmembrane alpha‐helical domain, extracellular beta‐fold region, and dynamic amphiphilic leucine‐rich disordered/protein‐binding motifs – exhibits similar functions requires further investigation. In a weighted gene co‐expression network analysis (WGCNA) of normal wild‐type (WT) mouse epidermal Langerhans cells (LC), where the LC population was divided into five distinct modules with screened differential genes, LSMEM1 ranked at the top of the module associated with “regulation of the citric acid cycle, respiratory electron transport chain, and cellular response to stress”,^[^ ^56^ ^]^ suggesting its potential involvement in these metabolic processes.

Emerging evidence highlights micropeptides' regulatory roles in glucose/lipid metabolism, making them promising therapeutic targets for severe metabolic disorders.^[^ ^57^ ^]^ With the advancement of technology, ribo‐seq, considered an effective detection method for small proteins,^[^ ^58^ ^]^ bridges the gap between RNA‐Seq and proteomics by providing translational information, enabling improved inference from the transcriptome to proteome.^[^ ^14^ ^]^ Micropeptides localized to cell membranes may exhibit enhanced stability and possibly resistance to rapid degradation.^[^ ^15^ ^]^ Assessing their practical applications requires technological innovation and interdisciplinary collaboration.^[^ ^59^, ^60^ ^]^

## 4 Summary of the Study

Our integrated transcriptomic and experimental analysis revealed key insights about LSMEM1:

1. This structurally unique micropeptide (featuring transmembrane α‐helical and extracellular β‐fold domains with amphiphilic/leucine‐rich motifs) shows multifunctional potential.
2. LSMEM1 is predominantly expressed in proximal tubule cells and podocytes, with detectable levels in parietal epithelial cells. Given their shared embryonic origin, LSMEM1's role in these lineages remains unclear, despite its marked upregulation in chronic kidney disease.
3. Knockout or silencing of LSMEM1 exacerbates lipid accumulation in the kidney, while its overexpression alleviates this condition.
4. LSMEM1 may function as a protective protein involved in multiple biological processes, including lipid metabolism.

## 5 Limitations of the Study

While this study provides the first transcriptional characterization of LSMEM1 under physiological conditions, several limitations should be acknowledged. The low abundance of podocytes and parietal epithelial cells in our samples constrained robust analysis of these populations, and our findings in proximal tubule cells represent only preliminary insights into LSMEM1's biological functions. Methodologically, we selected male mice for renal modeling due to the well‐documented renal protective effects of estrogen in females,^[^ ^61^, ^62^, ^63^ ^]^ focusing on tubulointerstitial fibrosis models where LSMEM1 expression was most prominent and analyzing the predominant PT cell population that showed strong LSMEM1 co‐localization and lipid metabolic pathway enrichment. While these methodological strategies have successfully established the preliminary association between LSMEM1 and lipid metabolic regulation, the exact molecular mechanisms underlying this relationship, particularly at both transcriptional (mRNA) and translational (protein) levels, await further elucidation. Furthermore, while observed expression differences in diabetic nephropathy models and LSMEM1's localization patterns in podocytes and parietal epithelial cells suggest broader functional significance across renal cell types and diseases, these aspects require systematic investigation. Additional limitations include the need for structural characterization of LSMEM1, determination of its subcellular organelle localization and elucidation of its degradation pathways, all warranted by its unique structural features. The current use of whole‐body knockout models and future development need cell‐specific knockout systems in both male and female mice model for more precise mechanistic studies. These limitations notwithstanding, our work establishes foundational knowledge about this novel micropeptide and provides a methodological framework for future investigations of similar newly discovered molecules.

## 6 Experimental Section

### Human Samples

The patient study was conducted according to the Second Helsinki Declaration and obtained necessary approval from the Ethics Committee for Human Research of Guangdong Provincial People's Hospital (Ethics No.KY‐N‐2022‐050). Normal controls and patients with pathologically confirmed CKD (including AKI to CKD phase, LN, IgAN, CIN, MN, DN, and HTN) treated at Guangdong Provincial People's Hospital were enrolled. Non‐tumor kidney cortex samples were obtained from patients undergoing partial or radical nephrectomy for renal mass at Guangdong Provincial People's Hospital. Informed consent was obtained from all patients and histologic sections were reviewed by a renal pathologist with laboratory data abstracted from medical records.

### Data Access

Raw and processed data are available from the Gene Expression Omnibus (GEO) (www.ncbi.nlm.nih.gov/geo/), series accession number: GSE180394.

### Animal Models

All animal experiments were conducted according to the guidelines of the Animal Research: Reporting of In Vivo Experiments (ARRIVE) and obtained necessary approval from the Ethics Committee for animal research of Guangdong Provincial People's Hospital (Ethics No.KY‐N‐2022‐050). Wild‐type (WT) male mice (background: C57/BL6J) were obtained from the Experimental Animal Center of Guangdong Academy of Medical Sciences. Male total Lsmem1 deletion (Lsmem1^−/−^, C57BL/6J background; 8–10 weeks old, 18–23 g) mice were provided by Shanghai Model Organisms Center. The animals were housed at the Specific Pathogen‐Free Laboratory Animal Center of Guangdong Academy of Medical Sciences with a 12 h light/12 h dark cycle, 60% humidity, and 25 °C. Mice had free access to water and food. After completion of experiments, mice were humanely sacrificed, and blood and kidney samples were collected. Tissue samples were fixed by immersion in OCT or 4% paraformaldehyde, then snap‐frozen in liquid nitrogen for subsequent experiments. For more detailed methodologies, please refer to the Supporting Information.

### Sample Processing and Cell Sorting

Fresh samples were stored in MACS Tissue Storage Solution (Miltenyi Biotec) on ice and immediately transferred to the laboratory. After washing with phosphate‐buffered saline (PBS), the samples were enzymatically digested to obtain single‐cell suspensions. The samples were minced into <1 mm^[^ ^3^ ^]^ pieces and digested with 5 mL of digestion buffer containing collagenase IV (2 mg mL^−1^; SigmaAldrich) and deoxyribonuclease I (1mg/ml; Sigma‐Aldrich) for 30 min at 37 °C. The resulting suspension was mixed with 5 mL of 2% fetal bovine serum (FBS)/PBS and centrifuged at 300g for 5 min at 4 °C to obtain the cell pellet. The cell pellet was then mixed with red blood cell lysis buffer (BD) for 3 min at room temperature and centrifuged at 300 g for 5 min at 4 °C. After this, the cells were incubated with antibodies for 30 min at 4 °C, followed by staining with 7‐aminoactinomycin D (7‐AAD) (eBioscience) before cell sorting.

### Single‐Cell Library Preparation and Sequencing

The Single‐Cell 3’ Library Kitv 3 (10× Genomics) was used for single cell transcriptome amplification and library preparation according to the manufacturer's instructions. The sorted single‐cell suspension was loaded on to a microfluidic chip from 10× Genomics to generate the cDNA library. Next, cDNA libraries were prepared and sequenced across six lanes on an Illumina NovaSeq 6000 system (Illumina Inc., SanDiego, CA, USA).

### Preprocessing of scRNA‐Seq Data

Raw sequencing FASTQ files were aligned to the GRCh38 reference genome using the cell ranger count function of CellRanger (10xGenomics) and the STAR algorithm to produce a gene expression library. The raw gene expression matrices were then processed using the Seurat R package. Only genes expressed in at least 10 cells in a sample were included. Low‐quality cells were removed based on the following criteria: fewer than 2000 unique molecular identifiers (UMIs), more than 3000 or fewer than 501 expressed genes, or over 10% of UMIs derived from the mitochondrial genome. Cell doublets were removed using the Doublet Finder R package. The single‐cell transcriptome expression matrices of the remaining high‐quality cells were integrated using the harmony R package, normalized to the total cellular UMI count, and scaled (scale factor = 1×104) by regressing out the total cellular UMI counts and percentage of mitochondrial genes. Highly variable genes (HVGs) were selected for Uniform Manifold Approximation and Projection (UMAP) and t‐distributed stochastic neighbor embedding (tSNE) dimension reduction, and visualization of gene expression.

### Determination of Cell Type

DEGs of each cell subcluster were identified using single R. Cell types and subtypes were annotated based on expression of known canonical marker genes. Cell subclusters with similar gene expression patterns were annotated as the same cell type.

### Enrichment Analysis

DEGs with |log_2_FC| >0.36 and adjusted *p* value <0.05 were used for GO/KEGG/GSEA enrichment analysis. The compare Cluster function in the cluster Profiler R package was used to find different enriched terms between distinct subclusters.

### Tyramide Signal Amplification (TSA) Technology

Kidney tissues were dissected, embedded in OCT compound (Sakura, 4583), and frozen immediately at −80 °C. Tissue sections were fixed in 4% paraformaldehyde at room temperature for 15 min. Using Multiplex fluorescence immunohistochemistry kits (Panovue, 10203100020) according to the manufacturer's instructions. Primary antibodies to LSMEM1 (Novus, NBP1‐81923), FATP2 (Proteintech, 68074‐1), ATP1A1 (Proteintech, 68735‐2), WT1 (Abcam, ab89901), and KIM1 (Abcam, ab78494) were added and incubated at 4°C overnight. Images were obtained on a confocal microscope (Zeiss, Oberkochen, Germany).

### Immunohistochemistry

The 2.5‐micron‐thick renal tissue sections were deparaffinized, rehydrated, and rinsed in distilled water. Antigen retrieval was performed using a pressure cooker with 1mM EDTA buffer (pH 8.0) for 3 min. Endogenous peroxidase activity was blocked by incubating the slides in 10% hydrogen peroxide in methanol for 10 min and nonspecific staining was inhibited with 5% bovine serum albumin for 30 min. After incubation with the LSMEM1 (Novus, NBP1‐81923) Rabbit IgG polyclonal antibody at 4 °C overnight, tissue sections were washed three times with PBS and incubated with the peroxidase (HRP)‐conjugated Enzyme‐labeled goat anti‐mouse/rabbit IgG polymer antibody (GT, GK600710) at room temperature for 60 min. The reaction was detected using HRP‐catalyzed diaminobenzidine (DAB) staining according to the manufacturer's instructions (GT, GK600710).

### Western Blot Analysis

Total protein was extracted from kidney tissue using radio‐immunoprecipitation assay (RIPA) lysate buffer (Thermo Fisher Scientific, Waltham, MA, USA), supplemented with phenylmethyl sulfonyl fluoride (Thermo Fisher Scientific, Waltham, MA, USA). Protein concentration was detected using a bicinchoninic acid (BCA) protein assay kit (Thermo Fisher Scientific, Waltham, MA, USA). In total, 20–100 µg of protein was resolved by 8%–16% SDS‐polyacrylamide gel electrophoresis and electro‐blotted onto polyvinylidene fluoride (PVDF) membranes (Thermo Fisher Scientific, Waltham, MA, USA). Membranes were incubated with primary antibodies, anti‐LSMEM1 and GAPDH (Proteintech, 60004‐1), at 4 °C overnight, then HRP‐conjugated secondary antibodies (Rabbit, Abcam, ab6721; Mouse, Abcam, ab6789), at room temperature for one hour. Antibody‐antigen complexes were detected using Western blotting chemiluminescence luminol reagent (Millipore). Signals were quantified by Image J software, defined as the ratio of target protein to GAPDH. For detection of endogenous LSMEM1 in knockdown experiments (Figure 6), larger sample volumes (40 µg protein/lane) were loaded to compensate for low basal expression levels. In overexpression studies (Figure S5, Supporting Information), lysates containing 3×Flag‐tagged LSMEM1 (adding ∼3 kDa to the predicted molecular weight) were loaded at 10 µg protein/lane due to higher expression abundance.

### TG/TC/HDL/LDL Measurement

TG/TC/HDL/LDL contents in kidney and HK‐2 cells were measured using quantification kits (Cayman Chemical, Ann Arbor, MI) according to the manufacturer's instructions.

### Statistics

Statistical analyses were performed using GraphPad Prism software package. One‐way or two‐way ANOVA followed by Tukey's test was used to assess differences among groups. The unpaired t‐test was used to compare differences between two groups. Results are expressed as mean ± standard deviation (SD) or number (percentage) for categorical variables. Data points that significantly deviate from the overall dataset pattern (exceeding ±3 standard deviations from the mean) are defined as outliers. All tests were two‐sided, and *p* < 0.05 was considered statistically significant.

## Conflict of Interest

The authors declare no competing interests.

## Author Contributions

P.L. and H.Z. contributed equally to this study. X.B. contributed to the experimental design and provided constructive suggestions. P.L., S.Y., and H.Z. performed in vivo animal studies and in vitro experiments. H.X., H.J., J.L., Y.J. and D.W. collected clinical samples and data. X.B. interpreted the data, wrote the manuscript, and approved the final version for publication.

## Supporting information



Supporting Information

Supporting Information

Supporting Data

## Data Availability

The data that support the findings of this study are available in the supplementary material of this article.

## References

[1] C. A. Makarewich , E. N. Olson , Trends Cell Biol. 2017, 27, 685. 28528987 10.1016/j.tcb.2017.04.006 PMC5565689

[2] M. E. Sousa , F. M. H. Micropeptide , PLoS Genet. 2018, 14, 1007764. 10.1371/journal.pgen.1007764 PMC6292567 30543625

[3] R. Vitorino , S. Guedes , F. Amado , M. Santos , N. Akimitsu , http://ras.smu.edu.cn/s/com/springer/link/G.https/article/10.1007/s00018‐020‐03740‐3?;x‐chain‐id=9fg6t6l4hfcw (accessed: May 2024). 10.1007/s00018-020-03740-3 PMC11073438 33507325

[4] A. Kvist‐Hansen , H. Kaiser , X. Wang , M. Krakauer , P. M. Gørtz , B. D. McCauley , C. Zachariae , C. Becker , P. R. Hansen , L. Skov , Int. J. Mol. Sci. 2021, 22, 10818. 34639156 10.3390/ijms221910818 PMC8509817

[5] Y. Li , H. Wang , Y. Pan , S. Wang , Z. Zhang , H. Zhou , M. Xu , X. Liu , Front. Endocrinol. 2023, 14, 1125299. 10.3389/fendo.2023.1125299 PMC10151815 37143720

[6] B. J. Whittle , O. G. Izuogu , H. Lowes , D. Deen , A. Pyle , J. Coxhead , R. A. Lawson , A. J. Yarnall , M. S. Jackson , M. Santibanez‐Koref , G. Hudson , NPJ Parkinsons Dis. 2024, 10, 25. 38245550 10.1038/s41531-024-00636-y PMC10799891

[7] M. Alradhi , S. Wen , M. Safi , A. Al‐danakh , H. Wang , A. Shopit , M. Sun , B. Fan , X. Li , Cancer Med. 2022, 12, 3931. 36779496 10.1002/cam4.5121 PMC9972163

[8] M. Vendruscolo , M. Fuxreiter , J. Mol. Biol. 2022, 434, 167201. 34391803 10.1016/j.jmb.2021.167201

[9] A. Hatos , S. C. E. Tosatto , M. Vendruscolo , M. Fuxreiter , Nucleic Acids Res. 2022, 50, 337. 10.1093/nar/gkac386 PMC9252777 35610022

[10] M. Hardenberg , A. Horvath , V. Ambrus , M. Fuxreiter , M. Vendruscolo , Proc. Natl. Acad. Sci. U.S.A. 2020, 117, 33254. 33318217 10.1073/pnas.2007670117 PMC7777240

[11] M. E. Oates , P. Romero , T. Ishida , M. Ghalwash , M. J. Mizianty , B. Xue , Z. Dosztányi , V. N. Uversky , Z. Obradovic , L. Kurgan , A. K. Dunker , J. Gough , Nucleic Acids Res. 2013, 41, 508. 10.1093/nar/gks1226 PMC3531159 23203878

[12] Y. F. Li , X. Pan , H. B. Shen , bioRxiv 2025, 6, 10.1016/j.patter.2025.101262.

[13] K. C. Chou , H. B. Shen , Nat. Protoc. 2008, 3, 153. 18274516 10.1038/nprot.2007.494

[14] V. Olexiouk , G. Menschaert , Proteogenomics 2016, 49. 10.1007/978-3-319-42316-6_4 27686805

[15] H. Zhou , Y. Wu , J. Cai , D. Zhang , D. Lan , X. Dai , S. Liu , T. Song , X. Wang , Q. Kong , Z. He , J. Tan , J. Zhang , Cancer Cell Int. 2024, 24, 134. 38622617 10.1186/s12935-024-03281-w PMC11020647

[16] H. Ryu , A. Fuwad , S. Yoon , H. Jang , J. C. Lee , S. M. Kim , T.‐J. Jeon , Int. J. Mol. Sci. 2019, 20, 1437. 30901910 10.3390/ijms20061437 PMC6472214

[17] E. A. Klimanova , S. V. Sidorenko , P. A. Abramicheva , A. M. Tverskoi , S. N. Orlov , O. D. Lopina , Int. J. Mol. Sci. 2020, 21, 7992. 33121152 10.3390/ijms21217992 PMC7662270

[18] A. M. Vargason , A. C. Anselmo , S. Mitragotri , Nat. Biomed. Eng. 2021, 5, 951. 33795852 10.1038/s41551-021-00698-w

[19] S. Zeng , Y. Ye , H. Xia , J. Min , J. Xu , Z. Wang , Y. Pan , X. Zhou , W. Huang , Eur. J. Med. Chem. 2023, 261, 115793. 37708797 10.1016/j.ejmech.2023.115793

[20] P. Zhao , S. Song , Z. He , G. Dai , D. Liu , J. Shen , T. Asakawa , M. Zheng , H. Lu , BST 2023, 17, 503. 10.5582/bst.2023.01285 38072446

[21] A. Hultgårdh‐Nilsson , J. Borén , S. Chakravarti , J. Intern. Med. 2015, 278, 447. 26477596 10.1111/joim.12400 PMC4616156

[22] A. Tan , R. Prasad , C. Lee , J. E. hoon , Cell Death Differ. 2022, 29, 1433. 35739255 10.1038/s41418-022-01028-6 PMC9345944

[23] A. Sahgal , V. Uversky , V. Davé , Methods 2023, 220, 38. 37890707 10.1016/j.ymeth.2023.10.009

[24] A. A. Bergen , S. Kaing , J. B. Brink , The Netherlands Brain Bank , T. G. Gorgels , S. F. Janssen , BMC Genomics 2015, 16, 956. 26573292 10.1186/s12864-015-2159-z PMC4647590

[25] B. J. Kim , Y. Kim , D. H. Youn , J. J. Park , J. K. Rhim , H. C. Kim , K. Kang , J. P. Jeon , Sci. Rep. 2020, 10, 11419. 32651463 10.1038/s41598-020-68325-3 PMC7351711

[26] S. Zhang , H. Zou , X. Zou , J. Ke , B. Zheng , X. Chen , X. Zhou , J. Wei , J. Mol. Neurosci. 2023, 73, 316. 37133759 10.1007/s12031-023-02108-z PMC10200785

[27] A. R. Henderson , Q. Wang , B. Meechoovet , A. L. Siniard , M. Naymik , M. De Both , M. J. Huentelman , R. J. Caselli , E. Driver‐Dunckley , T. Dunckley , Front. Genet. 2021, 12, 640266. 33981329 10.3389/fgene.2021.640266 PMC8107387

[28] H. Li , G. Hong , M. Lin , Y. Shi , L. Wang , F. Jiang , F. Zhang , Y. Wang , Z. Guo , Sci. Rep. 2017, 7, 14027. 29070791 10.1038/s41598-017-13700-w PMC5656592

[29] W. M. Brandler , D. Antaki , M. Gujral , A. Noor , G. Rosanio , T. R. Chapman , D. J. Barrera , G. N. Lin , D. Malhotra , A. C. Watts , L. C. Wong , J. A. Estabillo , T. E. Gadomski , O. Hong , K. V. F. Fajardo , A. Bhandari , R. Owen , M. Baughn , J. Yuan , T. Solomon , A. G. Moyzis , M. S. Maile , S. J. Sanders , G. E. Reiner , K. K. Vaux , C. M. Strom , K. Zhang , A. R. Muotri , N. Akshoomoff , S. M. Leal , et al., Am. J. Hum. Genet. 2016, 98, 667. 27018473 10.1016/j.ajhg.2016.02.018 PMC4833290

[30] J. R. Perkins , A. Antunes‐Martins , M. Calvo , J. Grist , W. Rust , R. Schmid , T. Hildebrandt , M. Kohl , C. Orengo , S. B. McMahon , D. L. Bennett , Mol. Pain. 2014, 10, 7. 24472155 10.1186/1744-8069-10-7 PMC4021616

[31] G. Kildisiute , W. M. Kholosy , M. D. Young , K. Roberts , R. Elmentaite , S. R. van Hooff , C. N. Pacyna , E. Khabirova , A. Piapi , C. Thevanesan , E. Bugallo‐Blanco , C. Burke , L. Mamanova , K. M. Keller , K. P. S. Langenberg‐Ververgaert , P. Lijnzaad , T. Margaritis , F. C. P. Holstege , M. L. Tas , M. H. W. A. Wijnen , M. M. van Noesel , I. del Valle , G. Barone , R. van der Linden , C. Duncan , J. Anderson , J. C. Achermann , M. Haniffa , S. A. Teichmann , D. Rampling , et al., Sci. Adv. 2021, 7, 3311. 10.1126/sciadv.abd3311 PMC7864567 33547074

[32] H. Razzaghi , M. Khabbazpour , Z. Heidary , M. Heiat , Z. Shirzad Moghaddam , P. Derogar , A. Khoncheh , M. Zaki‐Dizaji , Arch. Iran Med. 2023, 26, 447. 38301107 10.34172/aim.2023.68 PMC10685733

[33] P. Wang , W. Zhao , H. Cao , Front. Genet. 2022, 13, 904168. 35719389 10.3389/fgene.2022.904168 PMC9198283

[34] W. Zhang , R. Sun , Y. Zhang , R. Hu , Q. Li , W. Wu , X. Cao , J. Zhou , J. Pei , P. Yuan , FEBS Open Bio 2021, 11, 3032. 10.1002/2211-5463.13290 PMC8564099 34496154

[35] M. Giriyappagoudar , B. Vastrad , R. Horakeri , C. Vastrad , Bioinform. Biol. Insights 2023, 17, 11779322231186719. 37529485 10.1177/11779322231186719 PMC10387711

[36] J. Tu , J. Jin , X. Chen , L. Sun , Z. Cai , Front. Immunol. 2022, 13, 868983. 35663995 10.3389/fimmu.2022.868983 PMC9159786

[37] F. Gao , Y. Yao , Y. Zhang , J. Tian , Front. Genet. 2019, 10, 827. 31572443 10.3389/fgene.2019.00827 PMC6753977

[38] J. B. Freij , M. S. Fu , C. M. De Leon Rodriguez , A. Dziedzic , A. E. Jedlicka , Q. Dragotakes , D. C. P. Rossi , E. H. Jung , C. Coelho , A. Casadevall , Infect. Immun. 2018, 86, 00946. 10.1128/IAI.00946-17 PMC6013651 29712729

[39] N. Gomez‐Lopez , R. Romero , J. Galaz , G. Bhatti , B. Done , D. Miller , C. Ghita , K. Motomura , M. Farias‐Jofre , E. Jung , R. Pique‐Regi , S. S. Hassan , T. Chaiworapongsa , A. L. Tarca , Biol. Reprod. 2021, 106, 185. 10.1093/biolre/ioab197 PMC8897989 34686873

[40] W. Wei , N. Ma , X. Fan , Q. Yu , X. Ci , Free Radical Biol. Med. 2020, 158, 1. 32663513 10.1016/j.freeradbiomed.2020.06.025

[41] X. Liu , X. Zhao , X. Duan , X. Wang , T. Wang , S. Feng , H. Zhang , C. Chen , G. Li , Experim. Therap. Med. 2021, 21, 321. 10.3892/etm.2021.9752 PMC7903474 33732294

[42] C. Kuppe , K. Leuchtle , A. Wagner , N. Kabgani , T. Saritas , V. G. Puelles , B. Smeets , S. Hakroush , J. van der Vlag , P. Boor , M. Schiffer , H.‐J. Gröne , A. Fogo , J. Floege , M. J. Moeller , Kidney Int. 2019, 96, 80. 31029503 10.1016/j.kint.2019.01.037 PMC7292612

[43] L. W. Bin , H. G. Rui , L. B. Li , H. Hu , J. Geng , H. Rui , C. Gao , Y. Huang , G. Huo , J. Mao , C. Lu , A. Xu , Kidney Int. 2023, 104, 108. 37100348 10.1016/j.kint.2023.03.036

[44] K. S. Collins , M. T. Eadon , Y.‐H. Cheng , D. Barwinska , R. Melo Ferreira , T. W. McCarthy , D. Janosevic , F. Syed , B. Maier , T. M. El‐Achkar , K. J. Kelly , C. L. Phillips , T. Hato , T. A. Sutton , P. C. Dagher , Cells 2022, 11, 1166. 35406730 10.3390/cells11071166 PMC8997785

[45] Y. Muto , E. E. Dixon , Y. Yoshimura , H. Wu , K. Omachi , N. Ledru , P. C. Wilson , A. J. King , N. Eric Olson , M. G. Gunawan , J. J. Kuo , J. H. Cox , J. H. Miner , S. L. Seliger , O. M. Woodward , P. A. Welling , T. J. Watnick , B. D. Humphreys , Nat. Commun. 2022, 13, 1. 36310237 10.1038/s41467-022-34255-z PMC9618568

[46] Y. Muto , P. C. Wilson , N. Ledru , H. Wu , H. Dimke , S. S. Waikar , B. D. Humphreys , Nat. Commun. 2021, 12, 2190. 33850129 10.1038/s41467-021-22368-w PMC8044133

[47] A. Mitrofanova , S. Merscher , A. Fornoni , Nat. Rev. Nephrol. 2023, 19, 629. 37500941 10.1038/s41581-023-00741-w PMC12926870

[48] C. Li , H. Cheng , B. K. Adhikari , S. Wang , N. Yang , W. Liu , J. Sun , Y. Wang , Front. Endocrinol. 2022, 13, 820002. 10.3389/fendo.2022.820002 PMC8959308 35355561

[49] F. A. Chapman , D. Nyimanu , J. J. Maguire , A. P. Davenport , D. E. Newby , N. Dhaun , Nat. Rev. Nephrol. 2021, 17, 840. 34389827 10.1038/s41581-021-00461-z PMC8361827

[50] C. Attané , C. Foussal , S. Le Gonidec , A. Benani , D. Daviaud , E. Wanecq , R. Guzmán‐Ruiz , C. Dray , V. Bezaire , C. Rancoule , K. Kuba , M. Ruiz‐Gayo , T. Levade , J. Penninger , R. Burcelin , L. Pénicaud , P. Valet , I. Castan‐Laurell , Diabetes 2012, 61, 310. 22210322 10.2337/db11-0100 PMC3266414

[51] J. Bergwik , A. Kristiansson , M. Allhorn , M. Gram , B. Åkerström , Front. Physiol. 2021, 12, 645650. 33746781 10.3389/fphys.2021.645650 PMC7965949

[52] Trends Genet. 2025, 41, 107.39753408

[53] Y. Chen , L. Ho , V. Tergaonkar , Cancer Lett. 2021, 500, 263. 33157158 10.1016/j.canlet.2020.10.038

[54] J. A. Olzmann , P. Carvalho , Nat. Rev. Mol. Cell Biol. 2019, 20, 137. 30523332 10.1038/s41580-018-0085-z PMC6746329

[55] L. Chen , M.‐L. Sha , F.‐T. Chen , C.‐Y. Jiang , D. Li , C.‐L. Xu , D.‐S. Pan , Z.‐J. Xu , Q.‐L. Tang , S.‐J. Xia , L.‐H. Sun , G.‐J. Fan , Y. Shao , Free Radical Biol. Med. 2023, 195, 132. 36584797 10.1016/j.freeradbiomed.2022.12.096

[56] Z. K. Tuong , S. W. Lukowski , Q. H. Nguyen , J. Chandra , C. Zhou , K. Gillinder , A. A. Bashaw , J. R. Ferdinand , B. J. Stewart , S. M. Teoh , S. J. Hanson , K. Devitt , M. R. Clatworthy , J. E. Powell , I. H. Frazer , iScience 2021, 24, 103326. 34805788 10.1016/j.isci.2021.103326 PMC8586807

[57] Y. Lu , Y. Ran , H. Li , J. Wen , X. Cui , X. Zhang , X. Guan , M. Cheng , J. Zhejiang. Univ. Sci. B 2023, 24, 1106. 38057268 10.1631/jzus.B2300128 PMC10710913

[58] J. Yang , H. Zhuang , J. Li , A. B. Nunez‐Nescolarde , N. Luo , H. Chen , A. Li , X. Qu , Q. Wang , J. Fan , X. Bai , Z. Ye , B. Gu , Y. Meng , X. Zhang , D. Wu , Y. Sia , X. Jiang , W. Chen , A. N. Combes , D. J. Nikolic‐Paterson , X. Yu , J. Clin. Invest. 2024, 134. 10.1172/JCI178392 PMC11093611 38625739

[59] C. Isert , K. Atz , G. Schneider , Curr. Opin. Struct. Biol. 2023, 79, 102548. 36842415 10.1016/j.sbi.2023.102548

[60] J. Abramson , J. Adler , J. Dunger , R. Evans , T. Green , A. Pritzel , O. Ronneberger , L. Willmore , A. J. Ballard , J. Bambrick , S. W. Bodenstein , D. A. Evans , C.‐C. Hung , M. O'Neill , D. Reiman , K. Tunyasuvunakool , Z. Wu , A. Zemgulyte , E. Arvaniti , C. Beattie , O. Bertolli , A. Bridgland , A. Cherepanov , M. Congreve , A. I. Cowen‐Rivers , A. Cowie , M. Figurnov , F. B. Fuchs , H. Gladman , R. Jain , et al., Nature 2024, 630, 493. 38718835 10.1038/s41586-024-07487-w PMC11168924

[61] D. Laouari , P. Vergnaud , T. Hirose , M. Zaidan , M. Rabant , C. Nguyen , M. Burtin , C. Legendre , P. Codogno , G. Friedlander , D. Anglicheau , F. Terzi , Kidney Int. 2022, 102, 78. 35337891 10.1016/j.kint.2022.02.027

[62] S. A. van Eeghen , L. Pyle , P. Narongkiatikhun , Y. J. Choi , W. Obeid , C. R. Parikh , T. G. Vosters , I. G. M. van Valkengoed , M. M. Krebber , D. J. Touw , M. den Heijer , P. Bjornstad , D. H. van Raalte , N. J. Nokoff , J. Clin. Invest. 2025, 135, 190850. 10.1172/JCI190850 PMC12043095 40193283

[63] J. J. Carrero , M. Hecking , N. C. Chesnaye , K. J. Jager , Nat. Rev. Nephrol. 2018, 14, 151. 29355169 10.1038/nrneph.2017.181

