# Supplementary figures and images for: Single‐Cell Dissection of the Biological Function and Molecular Features Underlying the Micropeptide LSMEM1 in Kidney
Source: Adv Sci (Weinh). 2025 Aug 27;12(48):e07713. doi: 10.1002/advs.202507713 (PMC12752587; doi:10.1002/advs.202507713)

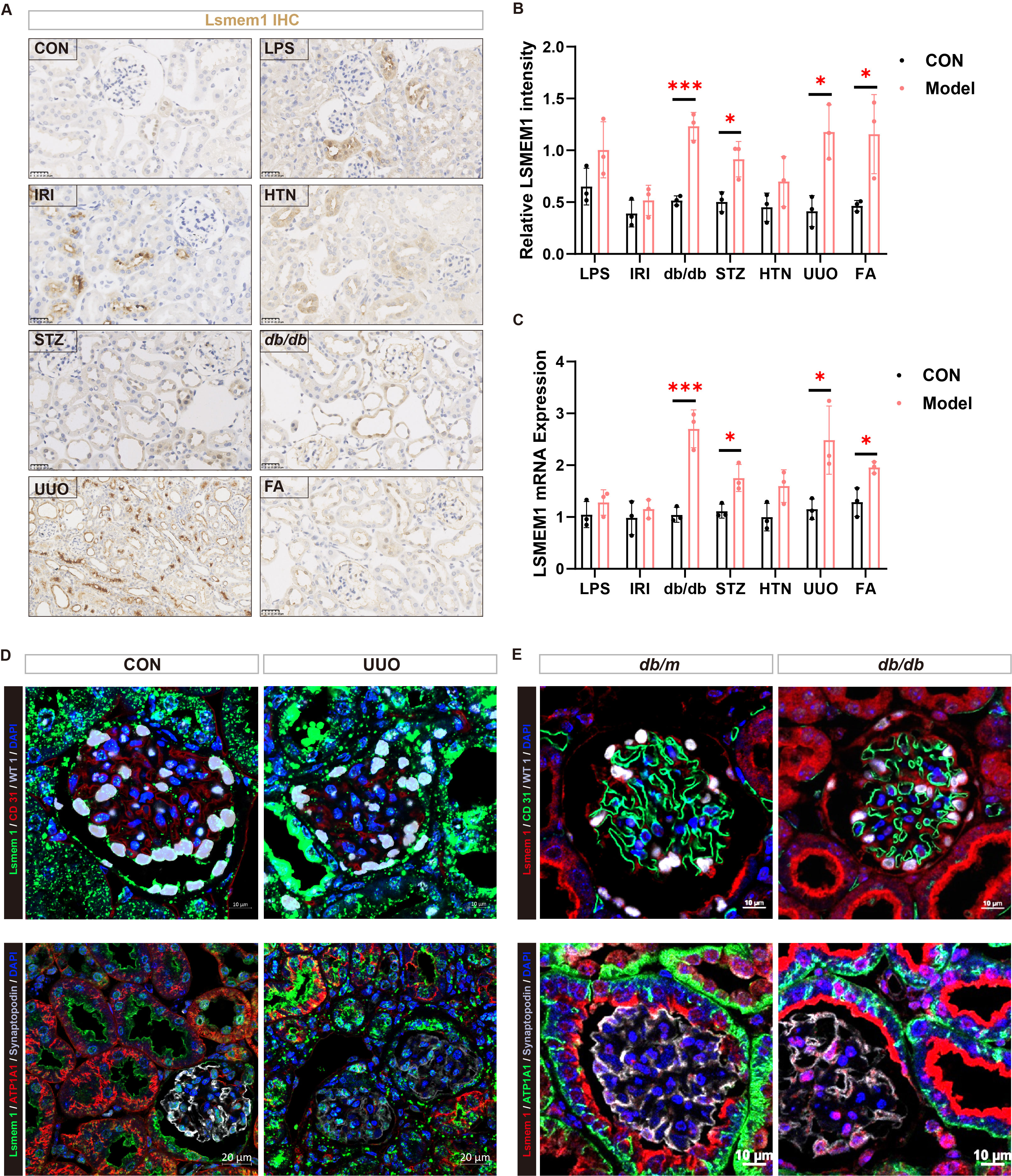

Supplement: Supplementary file 1 — Supporting Information [file ADVS-12-e07713-s003.jpg]
