# Supplementary material for: Single‐Cell Dissection of the Biological Function and Molecular Features Underlying the Micropeptide LSMEM1 in Kidney
Source: Adv Sci (Weinh). 2025 Aug 27;12(48):e07713. doi: 10.1002/advs.202507713 (PMC12752587; doi:10.1002/advs.202507713)
Supplement: Supplementary file 2 — Supporting Information [file ADVS-12-e07713-s001.docx]

**Supplementary Tables**

**Content:**

**Supplementary Table S1: Clinical data of patients**

**Supplementary Table S2: biological parameters of mice**

**Supplementary Table S3: The molecular information of LSMEM1**

**Supplementary Table S4: Prediction of subcellular localization of LSMEM1 protein**

**Supplementary Table S5: Potential segment important for nuclear transport**

**Supplementary Table S6: Representative markers of kidney cell types**

**Supplementary Table S7: Biological parameters of mice in LSMEM1^-/-^ mice**

**Supplementary Table S1: Clinical parameters of CKD patients and controls**

| **Number** | **Age** | **Gender** | **BMI** | **SBP (mmHg)** | **Scr (μmol/L)** | **BUN (mmol/L)** | **UACR**  **(mg/g)** |
| --- | --- | --- | --- | --- | --- | --- | --- |
| CON NO.1-TBMN | 35 | Male | 25.35 | NA | 108.30 | 5.70 | NA |
| CON NO.2-TBMN | 53 | Female | 24.14 | 151 | 42.60 | 4.10 | NA |
| CON NO.3 | 52 | Female | 22.69 | 120 | 83.00 | 6.64 | NA |
| AKI NO.1 | 23 | Female | 26.60 | 97 | 647.85 | 15.51 | 6302.80 |
| AKI NO.2 | 74 | Female | 25.02 | 123 | 64.77 | 7.83 | 1844.24 |
| AKI NO.3 | 21 | Male | 27.47 | 77 | 67.67 | 5.22 | 5456.44 |
| IgAN NO.1 | 54 | Male | 23.01 | 93 | 239.50 | 13.28 | 711.65 |
| IgAN NO.2 | 42 | Male | 23.50 | 94 | 201.90 | 14.04 | 99.07 |
| IgAN NO.3 | 36 | Male | 22.41 | 98 | 215.29 | 16.27 | 213.65 |
| MN NO.1 | 40 | Male | 17.72 | 118 | 94.40 | 4.83 | 1887.89 |
| MN NO.2 | 39 | Female | 25.96 | 131 | 53.88 | 3.80 | 475.04 |
| MN NO.3 | 38 | Female | 20.93 | 112 | 149.15 | 2.12 | 25.22 |
| HTN NO.1 | 49 | Female | 29.90 | 105 | 193.48 | 9.23 | 11.53 |
| HTN NO.2 | 50 | Female | 26.48 | 124 | 376.25 | 18.48 | 1207.64 |
| HTN NO.3 | 59 | Male | 30.12 | 117 | 186.88 | 11.7 | 419.48 |
| LN NO.1 | 14 | Female | 25.83 | 79 | 280.00 | 7.09 | 1298.46 |
| LN NO.2 | 24 | Female | 21.24 | 124 | 945.68 | 19.79 | 6004.09 |
| LN NO.3 | 23 | Male | 21.90 | 113 | 645.57 | 7.48 | 4525.34 |
| CIN NO.1 | 28 | Male | 30.16 | 96 | 224.33 | 7.09 | 853.24 |
| CIN NO.2 | 19 | Male | 17.31 | 77 | 405.02 | 19.79 | 8.36 |
| CIN NO.3 | 67 | Male | 20.31 | 113 | 63.55 | 7.48 | NA |
| DN NO.1 | 62 | Male | 23.42 | 123 | 176.52 | 15.29 | 827.96 |
| DN NO.2 | 58 | Male | 22.49 | 87 | 380.83 | 11.46 | 3541.41 |
| DN NO.3 | 57 | Female | 23.14 | 108 | 211.17 | 20.18 | 3715.00 |

CON: thin-basement membrane nephropathy (TBMN) and kidney tissues distant from kidney stone resection surgery; AKI: acute kidney injury; IgAN: IgA nephropathy; MN: membranous nephropathy; HTN: hypertensive nephropathy; LN: lupus nephritis; CIN: chronic interstitial nephritis; DN: diabetic nephropathy.

**Supplementary Table S2:** **biological parameters of mice**

|  | **Animals and treatments** | **Scr (μmol/L)** | **BUN (mmol/L)** | **SBP (mmHg)** | **UACR**  **(mg/g)** |
| --- | --- | --- | --- | --- | --- |
| **CON** (n=3) | C57BL/6 male mice (each group of mice had their own control); | 16.0162 | 11.05 | 79 | 78.94 |
| **LPS** (n=3) | C57BL/6 male mice;  LPS 10mg/kg i.p. 24h; | 442.00 | 88.72 | - | - |
| **IRI** (n=3) | C57BL/6 male mice;  Bilateral renal artery ischemia 30 min, reperfusion 48h; | 141.44 | 101.23 | - | - |
| **HTN** (n=3) | C57BL/6 male mice;  Subcutaneous back pump, AngⅡ1000 ng/kg/min 28d; | - | - | 121 | - |
| **STZ** (n=3) | C57BL/6 male mice;  Unilateral nephrectomy+STZ 50mg/kg i.p. 24w; | - | 38.75 | - | 446.76 |
| **db/db** (n=3) | Leptin receptor gene mutations male mice;  20w; | 105.48 | 35.80 | - | 521.34 |
| **UUO** (n=3) | C57BL/6 male mice;  unilateral ureteral obstruction 28d; | 145.86 | 16.13 | - | - |
| **FA** (n=3) | C57BL/6 male mice  FA (folic acid) 250 mg/kg i.p. 28d; | 126.00 | 17.66 | - | - |

**Supplementary Table S3: The molecular information of LSMEM1**

|  | **HUMAN** | **MUS** |
| --- | --- | --- |
| **Official Full Name** | leucine rich single-pass membrane protein 1 | |
| **Description** | Predicted to be integral component of membrane. | ①Predicted to be located in membrane.  ②Predicted to be integral component of membrane  ③Orthologous to human |
| **Also known as** | C7orf53 | Gm889 |
| **Length** | 131AA | 128AA |
| **Mass (Da)** | 14,462 | 14,186 |
| **Transmembrane region** | 62-84/86 | 62-84/86 |
| **Amino acid composition** |  |  |
| **Ala (A)** | 3 (2.3%) | 6 (4.7%) |
| **Arg (R)** | 5 (3.8%) | 6 (4.7%) |
| **Asn (N)** | 11 (8.4%) | 9 (7.0%) |
| **Asp (D)** | 11 (8.4%) | 9 (7.0%) |
| **Cys (C)** | 2 (1.5%) | 1 (0.8%) |
| **Gln (Q)** | 7 (5.3%) | 7 (5.5%) |
| **Glu (E)** | 4 (3.1%) | 7 (5.5%) |
| **Gly (G)** | 10 (7.6%) | 10 (7.8%) |
| **His (H)** | 2 (1.5%) | 3 (2.3%) |
| **Ile (I)** | 10 (7.6%) | 9 (7.0%) |
| **Leu (L)** | 19 (14.5%) | 18 (14.1%) |
| **Lys (K)** | 7 (5.3%) | 6 (4.7%) |
| **Met (M)** | 3 (2.3%) | 4 (3.1%) |
| **Phe (F)** | 6 (4.6%) | 6 (4.7%) |
| **Pro (P)** | 3 (2.3%) | 3 (2.3%) |
| **Ser (S)** | 11 (8.4%) | 10 (7.8%) |
| **Thr (T)** | 5 (3.8%) | 5 (3.9%) |
| **Trp (W)** | 0 (0.0%) | 0 (0.0%) |
| **Tyr (Y)** | 1 (0.8%) | 1 (0.8%) |
| **Val (V)** | 11 (8.4%) | 8 (6.2%) |
| **Pyl (O)** | 0 (0.0%) | 0 (0.0%) |
| **Sec (U)** | 0 (0.0%) | 0 (0.0%) |
| **Aliphatic index** | 112.98 | 105.08 |
| **GRAVY** | 0.017 | -0.105 |

GRAVY: Grand average of hydropathicity

The above information comes from the public databases of NCBI, Uniprot, InterPro, and psipred.

**Supplementary Table S4: Prediction of subcellular localization of LSMEM1 protein**

|  | **Query protein** | **Predicted location(s)** |
| --- | --- | --- |
| Hum-mPLoc 2.0 Computation Result | | |
|  | NP_001127940.1 leucine-rich single-pass membrane protein 1 [Homo sapiens] | Cytoplasm, Extracell, Nucleus |
|  | NP_872403.1 leucine-rich single-pass membrane protein 1 [Homo sapiens] | Cytoplasm, Extracell, Nucleus |
| Euk-mPLoc 2.0 Computation Result | | |
|  | NP_001028609.1 leucine-rich single-pass membrane protein 1 [Mus musculus] | Cell membrane, Cell wall, Cytoplasm, Endoplasmic reticulum, Extracell, Nucleus |

**Supplementary Table S5:** **Potential segment important for nuclear transport**

| **Position** | **Type** | **Length** | **Recommendation score** | **Entropy score** | **Segment** |
| --- | --- | --- | --- | --- | --- |
| 45-49 | NIA | 5 | 0.0075 | 2.32193 | EDKIP |
| 97-106 | NIA | 10 | 0.0067 | 3.12193 | RRLTAEGKDI |
| 67-71 | NIA | 5 | 0.00539 | 1.37095 | LLIVL |
| 23-32 | NIA | 10 | 0.00528 | 2.44644 | DSINDLNKLN |
| 59-63 | NIA | 5 | 0.00488 | 1.92193 | SRSLF |
| 15-19 | NIA | 5 | 0.00454 | 2.32193 | EDGKL |
| 73-77 | NIA | 5 | 0.00445 | 1.92193 | VSLAL |
| 115-119 | NIA | 5 | 0.004 | 2.32193 | MIVKR |
| 109-113 | NIA | 5 | 0.00364 | 2.32193 | LKRIN |
| 82-89 | NIA | 8 | 0.00357 | 2.75 | IFLIVQTG |

NIA: Nuclear Important Area

**Supplementary Table S6: Representative markers of kidney cell types**

| **Abbreviation** | **Cell type** | **Markers** |
| --- | --- | --- |
| ENDO | Endothelial | Cdh5, Kdr, Pecam1, Flt1, Emcn |
| PODO | Podocyte | Magi2, WT1, Nphs1, Nphs2, Podxl, Pla2r1 |
| MES | Mesangial cell | Itga8, Pdgfrb, Meis2, Piezo2, Ren |
| PEC | parietal epithelial cell | Vcam1, Cryab, Cfh, Aldh1a2 |
| PT | Proximal tubule | Pdzk1, Slc5a1, Slc5a2, slc34a1, Cyp4b1, Cyp7b1, Cubn, Havcr1, Cdh6, Ccl2, Prickle1, Tgfb2, Dcc, Lrp2 |
| TAL | Thick ascending limb | Umod, Banf2, Slc12a1, Sgcz, Tarm1, Egf, Cldn10, Cldn16 |
| DCT | Distal convoluted tubule | Banf2, Slc12a1, Nos1, Pappa2, Slc12a3, Trpm6, Scnn1g, Trpv5, Kcnh7 |
| CNT | Connecting tubule | Scnn1g, Trpv5, Calb1, Klhl3, Egfem1, Slc8a1, Slc14a2 |
| CD-PC | Collecting duct-principal cell | Frmpd4, Aqp4, Aqp2 |
| CD-IC | Collecting duct-intercalated cell | Slc26a4, Lsamp, Atp6v0d2, Slc4a1, Slc26a7, Cftr, Dgki, |
| FIB | Fibroblast | Acta2, Cald1, Fn1, Col1a1, Pdgfra, Fbln1 |
| T cell | T lymphocyte | Ptprc, Cd3e |
| B cell | B lymphocyte | Ptprc, Ms4a1 |
| MΦ | Macrophages | Ptprc, Cd14 |

**Supplementary Table S7: Biological parameters of mice in LSMEM1^-/-^ mice.**

|  | Body weight (g) | Body weight (g)- sacrifice | Kidney weight (g, R) | kidney weight (g, L) |
| --- | --- | --- | --- | --- |
| WT (n=6) | 22.218±0.132 | 22.474±0.410 | 0.131±0.008 | 0.122±0.006 |
| LSMEM1^-/-^ (n=6) | 21.877±1.304 | 21.408±1.341 | 0.133±0.012 | 0.137±0.012 |
| WT+UUO (n=8) | 21.087±1.457 | 20.889±1.056 | 0.171±0.010 | 0.101±0.008 |
| LSMEM1^-/-^+UUO (n=8) | 22.4±1.676 | 20.889±1.056 | 0.161±0.011 | 0.09±0.007 |

Body weight: Initial weight at procurement;

Body weight-sacrifice: Terminal weight at euthanasia;

Kidney weight (R/L): Weight of right/left kidney;

All data presented as mean with 95% confidence intervals. The serum creatinine (Cre) and blood urea nitrogen (BUN) levels are presented as bar graphs in Figure 5.
